# The Acute Impact of Ingestion of Sourdough and Whole-Grain Breads on Blood Glucose, Insulin, and Incretins in Overweight and Obese Men

**DOI:** 10.1155/2012/184710

**Published:** 2012-02-28

**Authors:** Anita Mofidi, Zachary M. Ferraro, Katherine A. Stewart, Hilary M. F. Tulk, Lindsay E. Robinson, Alison M. Duncan, Terry E. Graham

**Affiliations:** Department of Human Health and Nutritional Sciences, University of Guelph, Guelph, ON, Canada N1G 2W1

## Abstract

Consumption of whole-grain and sourdough breads is associated with improved glucose homeostasis. We examined the impact of commercial breads on biomarkers of glucose homeostasis in subjects at risk for glucose intolerance. In a randomized, crossover study, overweight or obese males ingested 11-grain, sprouted-grain, 12-grain, sourdough, or white bread on different occasions, matched for available carbohydrate (50 g) in part 1 (*n* = 12) and bread mass (107 g) in part 2 (*n* = 11), and blood glucose, insulin and glucose-dependent insulinotropic polypeptide (GIP) and glucagon-like peptide-1 (GLP-1) were determined for 3 h. In part 1, glucose response for sprouted-grain was lower than 11-grain, sourdough, and white breads. Insulin area under the curve (AUC) for sourdough and white was lower than 11-grain and sprouted-grain breads. GLP-1 response to sourdough was lower than all breads. In part 2, glucose and insulin AUC for sourdough was greater than 11-grain, sprouted-grain, and 12-grain breads. Sprouted-grain bread improved glycemia by lowering glucose response and increasing GLP-1 response. In overweight and obese men, the glycemic response to sprouted grain bread was reduced in both parts 1 and 2 while the other whole-grain test breads did not improve metabolic responses in the acute postprandial state.

## 1. Introduction

There is substantial interest in the role of dietary carbohydrate (CHO) in preventing and managing type 2 diabetes (T2D) [1]. In North America, bread is the predominant CHO-containing food, and consumption of white bread is 5 times that of whole wheat, rye, and other dark breads [2]. Replacing white bread with whole-grain breads is often recommended to improve glycemic control [3]. Epidemiologic studies have reported inverse associations between whole-grain consumption and the risk of T2D and cardiovascular disease [4–8], and clinical studies [9, 10] have reported beneficial effects of whole-grain consumption on the metabolic profile of subjects with impaired glycemic control. It has been suggested that the fiber content of whole-grain foods improves glucose/insulin metabolism by reducing the rate of CHO breakdown and absorption [11–13].

The incretin hormones, glucose-dependent insulinotropic polypeptide (GIP), and glucagon-like peptide-1 (GLP-1) are intimately involved in postprandial regulation of glucose homeostasis. It is estimated that approximately half of the postprandial insulin release in response to CHO ingestion is caused by these gut-derived hormones [14–17]. Thus, the magnitude of the incretin response is vital to both the acute insulinemic and glycemic responses to CHO ingestion. However, our understanding of the impact of different types of CHO on the incretin response is still in its infancy.

Previously, we showed that ingestion of whole-wheat and whole-wheat barley breads did not result in attenuated insulin responses compared with white bread [18]. Furthermore, sourdough white bread resulted in lower glucose and GLP-1 responses for two subsequent meal periods [18]. In our previous work, ultrafinely grounded whole-wheat flour was used rather than whole-grain flour. In addition, in order to equalize the amount of available CHO (50 g) across treatments, the bread mass consumed varied from 98 to 138 g resulting in higher energy, fat, protein, and fiber intake for the whole-wheat bread treatments [18]. Further study is needed to examine if bread mass influences the metabolic responses to bread.

 The sprouting treatment of cereal grains is reported to decrease starch content and increase the content and availability of vitamins, minerals, and antioxidants [19]. One clinical study reported improved glycemia following consumption of pregerminated brown rice, compared to white rice, in healthy and type T2D subjects [20]. The metabolic effect of breads baked with sprouted wheat flour has not been extensively studied.

The present investigation had a distinct applied nature and tested the hypothesis that consumption of laboratory-prepared sourdough bread and commercially available whole-grain and sprouted-grain breads would result in lower metabolic responses compared with commercial, white bread in subjects who are at risk for glucose intolerance and T2D. This hypothesis was tested using 2 approaches including normalizing consumption of breads according to available CHO (part 1) and bread mass (part 2).

## 2. Materials and Methods

The study protocol was approved by the University of Guelph Human Research Ethics Board and each subject provided written informed consent. Subjects were recruited from the Guelph, Ontario area through advertisement in local newspapers. Subjects were overweight or obese males (body mass index (BMI): 25–35 kg/m^2^), nonsmokers and had no history of gastrointestinal disease, gluten allergy, dyslipidemia, or diabetes. Subjects did not take medications (with the exception of antidepressants and/or antihypertensives) or natural health products. Potential subjects were screened for glucose intolerance and diabetes at a prestudy visit using a standard 2 h oral glucose tolerance test (OGTT) (Trutol Custom Laboratories Inc., Baltimore, MD). Subjects were excluded if they had impaired fasting plasma glucose (>6.1 mmol/L), impaired glucose tolerance (>7.8 mmol/L at 2 h), or impaired fasting insulin (>90 pmol/L).

### 2.1. General Protocol

Parts 1 and 2 of the investigation followed the same protocol, with the exception of the quantity of bread consumed. A single-blind, randomized crossover design was used with washout periods of at least 1 week between study days. Throughout the study, subjects were instructed to maintain their usual diet and lifestyle but were instructed to avoid alcohol, caffeine substances and strenuous physical activity 48 h prior to each study day and to report to the laboratory after an overnight (12 h) fast. Dietary records were kept for three days prior to each study day and in the evening before each study day, subjects were instructed to consume a standardized meal, consisting of vegetable lasagne (President's Choice Blue Menu Reduced Fat Vegetable Lasagne) and a cereal bar (Kellogg's Nutri-Grain Cereal Bar).

On each study day, a venous catheter was inserted into the forearm by a trained technician and kept patent for the duration of the experiment with a slow saline infusion. After collection of a fasting blood sample (time point −15 min), subjects consumed a serving of test bread with 250 mL of water within 15 min. The laboratory clock started when subjects commenced eating the bread, and after 15 min (time point zero) the second blood sample was collected. Subsequently, blood samples were collected at 15, 30, 45, 60, 90, 120, 150, and 180 min.

#### 2.1.1. Part 1: Acute Postprandial Effect of Ingestion of Breads Matched for Available Carbohydrate

Twelve overweight or obese males were recruited in part 1. The test breads were prepared to provide 50 g of available CHO which required portions of 151 g for 11-grain (whole-grain, with sourdough culture, Stone-mill Bakehouse Ltd., Scarborough, ON, Canada), 157 g of sprouted-grain (whole-grain, with sourdough culture, Stone-mill Bakehouse Ltd., Scarborough, ON, Canada), 107 g of sourdough white (as described previously [18] and baked at the Guelph Food Technology Centre at the University of Guelph), 122 g of 12-grain (whole-grain, Dempsters, Canada Bread Ltd., Brampton, ON, Canada), and 110 g of white bread (Wonder Bread, Weston Bakeries Ltd., Toronto, ON, Canada) (Table 1). Breads were sliced, decrusted and stored at −20°C until consumption. Before consumption, the bread slices were thawed in a microwave oven for 15 s and weighed.

#### 2.1.2. Part 2: Acute Postprandial Effect of Ingestion of Breads Matched for Mass

Eleven overweight or obese males completed part 2, 9 of whom also completed part 1. The same test breads studied in part 1 were prepared to provide a consistent portion of 107 g. This volume was selected as it allowed for a comparison between parts 1 and 2 for the ingestion of the same quantity of the sourdough bread. This resulted in portions of 35, 34, 50, 43, and 48 g of available CHO for 11-grain, sprouted-grain, sourdough white, 12-grain, and white bread, respectively (Table 2).

### 2.2. Blood Collection, Biochemical and Dietary Analysis

For analysis of blood glucose, blood samples were collected at all time points into vacutainers containing 72 USP units sodium heparin, immediately put on ice, and subsequently analyzed using a semiautomatic glucose analyzer (YSI 2300, Yellow Springs, OH, USA). For analysis of serum insulin, blood samples were collected at all time points into vacutainers without anticoagulants and centrifuged (1341 ×g for 10 min at 4°C). Serum supernatant was aliquoted and frozen at −20°C until analysis using a solid phase ^125^I radioimmunoassay (Coat-A-Count, Diagnostic Products Corporation, CA, USA) with an intra- and interassay variability of 5.2% and 7.3%, respectively.

 For analysis of the incretin hormones, blood samples were collected at all time points into ice-chilled tubes containing 10.8 mg K_2_EDTA, 1824 KIU aprotinin, and 10 *μ*L/mL blood dipeptidyl peptidase-4 inhibitor. Following centrifugation (1000 ×g for 15 min at 4°C), plasma was separated and stored at −80°C until analysis. Plasma GIP total concentrations were measured using a Human GIP (Total) ELISA kit (Linco Research Inc., St Charles, USA) with 100% crossreactivity to human intact GIP, GIP (1–42), and the N-terminally truncated metabolite, GIP (3–42). Intra- and interassay variability for GIP were 6.5% and 3.4%, respectively. Plasma GLP-1 total concentrations were measured by GLP-1 total RIA kit (Linco Research Inc., St Charles, USA) after extraction with 70% ethanol. The antibody used in this kit binds specifically with C-terminal portion of GLP-1, both amidated and nonamidated forms. Intra- and interassay variations for GLP-1 were 4.0% and 9.9%, respectively.

Food record data were analyzed for energy, macronutrients, cholesterol, and dietary fiber by using ESHA Food Processor program (version 9.5, Salem, OR, USA) and averaged across each 3-day food record.

### 2.3. Calculations and Statistical Analysis

 Incremental area under the curve (AUC) was determined for blood glucose, serum insulin, plasma GIP, and GLP-1 (GraphPad Prism, version 3.02, San Diego, CA, USA). Prism computes the incremental area under the curve by using the trapezoid rule. Time point −15 was used as the baseline, and values below the baseline were considered to be negative peaks. Insulin Sensitivity Index (ISI) was calculated using the method described by Matsuda and DeFronzo [21].

 All statistical analyses were performed using the Statistical Analysis System (SAS Institute Inc., version 9.1 Cary, NC, USA). Univariate analysis was used to examine the distribution of each variable, and logarithmic transformations were applied to data that was not normally distributed (specific variables are identified in data tables). Significance (*P* < 0.05) was tested by two-factor repeated measure analysis of variance (ANOVA) using a mixed model (treatment: fixed effect and subject: random effect) followed by the Tukey's test for multiple comparisons. Results are presented as mean ± SEM.

## 3. Results

### 3.1. Part 1: Acute Postprandial Effect of Ingestion Breads Matched for Available CHO

#### 3.1.1. Subjects

Twelve subjects (age: 54.9 ± 2.0 y, BMI: 29.1 ± 1.1 kg/m^2^, fasting blood glucose: 4.5 ± 0.1 mmol/L, fasting serum insulin: 50.8 ± 4.8 pmol/L) completed part 1 of the study.

#### 3.1.2. Blood Glucose

**Figure 1:**
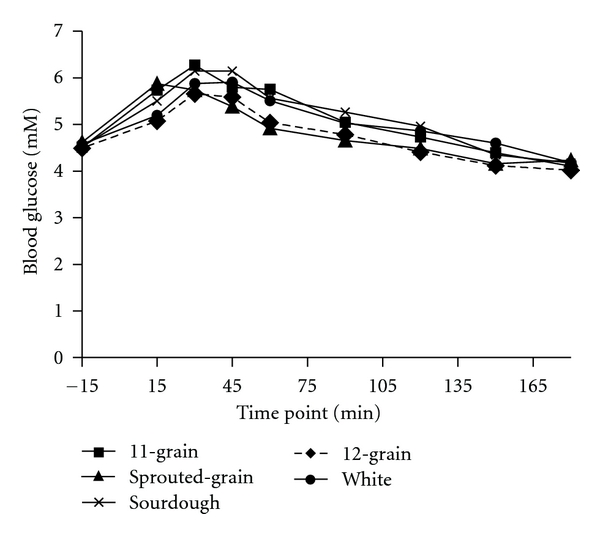
Fasting and postprandial glucose responses to the ingestion of 50 g available carbohydrate of the test breads. Data are means. Standard errors are not included for clarity, *n* = 12. Test bread was ingested after collection of fasting blood sample at time point −15 min. Significant overall treatment effects were found in glucose responses to the breads (Sprouted-grain bread was lower than 11-grain (*P* < 0.009), sourdough (*P* < 0.001), and white (*P* < 0.006) breads). Twelve-grain bread was lower than 11-grain (*P* < 0.04) and sourdough (*P* < 0.003) breads).

Significant overall treatment effects were found in glucose responses to the breads (Figure 1). Sprouted-grain bread was significantly lower than 11-grain (*P* < 0.009), sourdough (*P* < 0.001), and white (*P* < 0.006) breads. Furthermore, 12-grain bread was significantly lower than 11-grain (*P* < 0.04) and sourdough (*P* < 0.003) breads. Similarly, glucose incremental AUC for sprouted-grain bread was significantly lower than 11-grain (*P* < 0.007), sourdough (*P* < 0.004), and white (*P* < 0.05) breads (Table 3). Furthermore, glucose incremental AUC for 12-grain bread was significantly lower than 11-grain (*P* < 0.01) and sourdough (*P* < 0.009) breads (Table 3).

#### 3.1.3. Serum Insulin and Insulin Sensitivity

**Figure 2:**
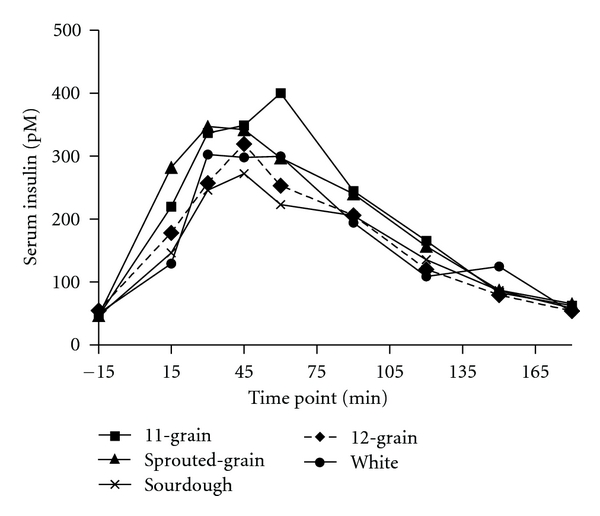
Fasting and postprandial insulin response to the ingestion of 50 g available carbohydrate of the test breads. Data are means. Standard errors are not included for clarity, *n* = 12. Test bread was ingested after collection of fasting blood sample at time point −15 min. Significant overall treatment effects were identified (11-grain bread was greater than sourdough (*P* < 0.005) and white (*P* < 0.03) breads).

Significant overall treatment effects were found in insulin responses to the breads with 11-grain bread being higher than sourdough (*P* < 0.005) and white (*P* < 0.03) breads (Figure 2). Furthermore, insulin incremental AUC for 11-grain and sprouted-grain breads was significantly (*P* < 0.05) greater than sourdough and white breads (Table 3). ISI was not significantly different among the breads (data not shown).

#### 3.1.4. Plasma GIP and GLP-1

**Figure 3:**
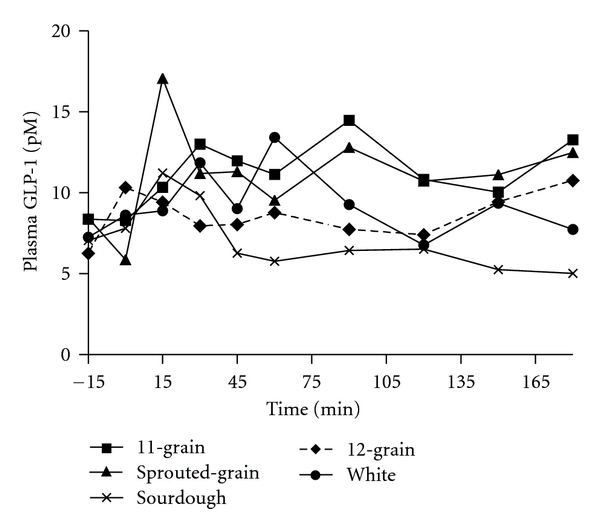
Fasting and postprandial GLP-1 responses to the ingestion of 50 g available carbohydrate of the test breads. Data are means. Standard errors are not included for clarity, *n* = 11. Test bread was ingested after collection of fasting blood sample at time point −15 min. Significant overall treatment effects were found (sourdough bread was lower than 11-grain (*P* < 0.0001), sprouted-grain (*P* < 0.0001), and white (*P* < 0.02) breads. 11-grain bread was greater than 12-grain (*P* < 0.03) and white (*P* < 0.03) breads. Sprouted-grain bread was greater than 12-grain (*P* < 0.009) and white (*P* < 0.05) breads).

Despite the difference in insulin responses, there was no significant overall treatment effect in GIP responses to the breads (data not shown). Similarly, bread treatment did not significantly affect GIP incremental AUC (Table 3). The significant differences in overall GLP-1 response to the breads did not correspond with those for insulin. The GLP-1 response to sourdough bread was lower than 11-grain (*P* < 0.0001), sprouted-grain (*P* < 0.0001), and white (*P* < 0.02) breads. Additionally, the GLP-1 response to 11-grain bread was greater than 12-grain (*P* < 0.03) and white (*P* < 0.03) breads, while the GLP-1 response to sprouted-grain bread was greater than 12-grain (*P* < 0.009) and white (*P* < 0.05) breads (Figure 3). Despite these differences, bread treatment did not significantly affect GLP-1 incremental AUC (Table 3).

### 3.2. Part 2: Acute Postprandial Effect of Ingestion of Breads Matched for Mass

#### 3.2.1. Subjects

Eleven subjects (age: 53.9 ± 1.7 y, BMI: 28.6 ± 0.7 kg/m^2^, fasting glucose: 4.6 ± 0.1 mmol/L, fasting insulin: 40.6 ± 5.7 pmol/L) completed part 2 of the study.

#### 3.2.2. Blood Glucose

Although there were no significant overall treatment effects in glucose responses to the breads, glucose incremental AUC for sourdough bread was significantly greater than 11-grain (*P* < 0.002), sprouted-grain (*P* < 0.01), 12-grain (*P* < 0.001), and white (*P* < 0.04) breads (Table 4).

#### 3.2.3. Serum Insulin and Insulin Sensitivity

**Figure 4:**
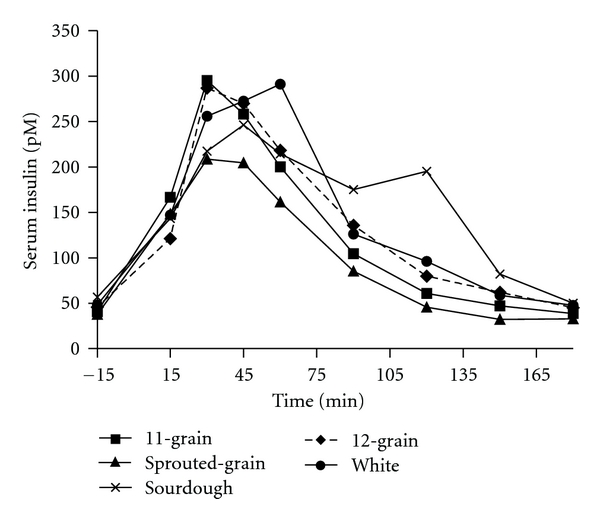
Fasting and postprandial insulin responses to the ingestion of a consistent amount of the test breads. Test bread was ingested after collection of fasting blood sample at time point −15 min. Data are means. Standard errors are not included for clarity, *n* = 11. Significant overall treatment effects were found in insulin response to the breads (sprouted-grain bread was lower than 12-grain (*P* < 0.03) bread, and 12-grain bread was lower than sourdough (*P* < 0.001) and white (*P* < 0.001) breads).

Significant overall treatment effects were found in insulin responses to the breads with sprouted-grain being lower than 12-grain (*P* < 0.03) bread and 12-grain bread being lower than sourdough (*P* < 0.001) and white (*P* < 0.001) breads (Figure 4). Insulin incremental AUC for 11-grain (*P* < 0.03), sprouted-grain (*P* < 0.05), and 12-grain (*P* < 0.0007) breads was significantly lower than sourdough bread. In addition, insulin incremental AUC for 12-grain was lower than white bread (*P* < 0.03) (Table 4). ISI was not significantly different among the breads (data not shown).

#### 3.2.4. Plasma GIP and GLP-1

**Figure 5:**
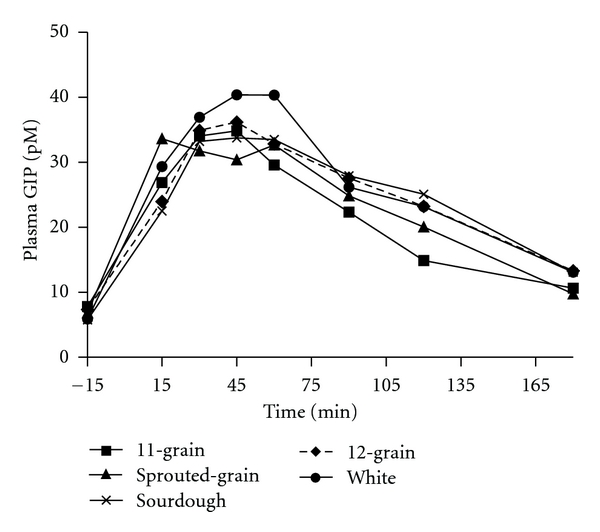
Fasting and postprandial GIP responses to the ingestion of a consistent amount of the test breads. Data are means. Standard errors are not included for clarity, *n* = 11. Test bread was ingested after collection of fasting blood sample at time point −15 min. A significant overall treatment effect was found (11-grain bread was lower than sourdough (*P* < 0.008) bread).

As in part 1, incretin responses did not correspond with the postprandial insulin response. Overall GIP response to 11-grain was lower than sourdough bread (*P* < 0.008) (Figure 5). GIP incremental AUC for 11-grain bread was significantly lower than sourdough (*P* < 0.03) and white (*P* < 0.001) breads (Table 4). Despite the modest (4.8 g) difference in the available CHO consumed, GIP incremental AUC for 12-grain was lower than white bread (*P* < 0.03) (Table 4).

**Figure 6:**
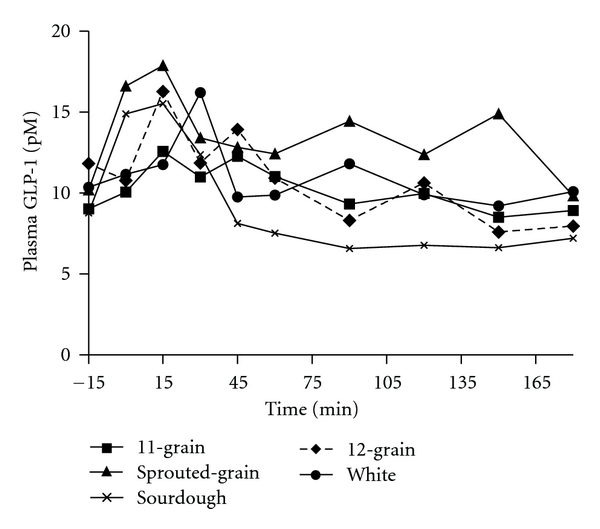
Fasting and postprandial GLP-1 responses to the ingestion of a consistent amount of the test breads. Data are means. Standard errors are not included for clarity, *n* = 10. Test bread was ingested after collection of fasting blood sample at time point −15 min. Significant overall treatment effects were found (sprouted-grain bread was greater than 11-grain (*P* < 0.008), sourdough (*P* < 0.001), 12-grain (*P* < 0.04), and white (*P* < 0.04) breads).

Similarly, GLP-1 response did not relate to the amount of available CHO consumed as the overall GLP-1 response to sprouted-grain bread was significantly greater than 11-grain (*P* < 0.008), sourdough (*P* < 0.001), 12-grain (*P* < 0.04), and white (*P* < 0.04) breads (Figure 6). GLP-1 incremental AUC for sprouted-grain was significantly greater than sourdough (*P* < 0.05) and 12-grain (*P* < 0.01) breads (Table 4).

## 4. Discussion

 The purpose of the current study was to determine the acute effects of breads of variable carbohydrate composition on postprandial glucose, insulin, and incretin responses in sedentary, overweight/obese males as this population represents a group that are at increased risk for T2D. We hypothesised that the sprouted-grain, whole-grain, and sourdough breads would lower the postprandial metabolic responses, in comparison to white bread, in both parts 1 and 2 of the study. The nature of the subjects and the testing of commercial breads given either in portions based on available CHO or volume are limitations to interpreting the data, but they also are a strength as the findings are very applicable to society. The key findings were that the sprouted grain bread reduced the glycemic responses in both parts of the study and also that generally the whole-grain breads did not have what could be interpreted as beneficial, metabolic responses. While some differences were observed in the incretin hormones, these did not correspond to the insulin responses.

 When 50 g of available CHO was ingested (part 1), the glucose response (overall and incremental AUC) to sprouted-grain bread was significantly less than 11-grain, sourdough, and white breads. Additionally, the glucose response (overall and incremental AUC) for 12-grain bread was significantly lower than sourdough and 11-grain breads. The favourable glucose responses to the sprouted-grain and 12-grain breads support our hypothesis. Greater fiber content in sprouted-grain and 12-grain breads (Table 1) may explain the lowered glycemia following their ingestion compared to white and sourdough breads. Dietary fiber is reported to attenuate glycemic response through its physical action in the gut which lowers the rate of CHO digestion and absorption [11, 12, 21–23]. However, the glucose-lowering effect of cereal fiber has been attributed primarily to soluble fiber [12, 22, 24], and in the present study, the fiber content of the sprouted-grain and 12-grain breads was predominantly insoluble fiber, suggesting that soluble fiber may not be the only component responsible for improving glycemia. Other nutrients and components in the sprouted-grain and 12-grain breads may also have positive health-related effects. It has been suggested that the sprouting treatment of cereal grains increases the content and availability of vitamins, minerals, and antioxidants [19], and whole-grains are known to contain higher amounts of vitamins, minerals, antioxidants, and phytochemicals. The presence of micronutrients such as magnesium, vitamin E, antioxidants, phenolic compounds, and phytoestrogens may act synergistically to lower glycemia [6–9, 25, 26].

The lack of significant difference in postprandial glucose response between the 11-grain and white bread was unexpected. It should be noted that there are several factors influencing the metabolic responses to breads including the flour particle size, kneading protocol, leavening process, and baking procedure [27–31], but we are currently unable to identify which specific factor may have accounted for the findings in the present study. A strength of this study is its applicability due to the use of commercially prepared breads, but this also presents a limitation as detailed information regarding ingredients (i.e., the grain/flour structure and proportion contribution to each bread) and processing techniques are not available. Furthermore, although we accept that sample size may be another limitation, our results strongly suggest that there are no acute metabolic differences among the other breads. However, this does not mean there are no benefits in consuming these breads rather that any of the benefits are not obvious within the few hours that we studied. In fact, large epidemiological studies show an inverse relationship with whole grain intake and risk of obesity, diabetes, and cardiovascular disease. A large study examining almost 43000 people for up to 12 years found that a diet high in whole grains was inversely associated with type 2 diabetes risk [5]. Although the physiological mechanisms remain unclear, the postprandial response to dietary fiber remains a promising mediator of improved health. Conversely, a small randomized crossover study with 30 subjects by Andersson and colleagues [32] aligns with our results suggesting a lack of a favorable postprandial metabolic response to whole grain when compared to refined meals in those who are healthy and slightly overweight. Overall, there is paucity of information on the metabolic responses to breads of varying carbohydrate in overweight and obese men, and investigation with larger sample sizes is warranted to better understand the biological mediators of glycemic control.

While it is possible that any positive effect of the 11-grain bread would be apparent only after a chronic intervention, our findings clearly highlight that whole-grain breads are not the same. Eleven-grain bread was prepared with sourdough culture and contained high amount of fiber and did not improve glycemia; this finding suggests that one cannot generalize across whole-grain products, and the metabolic responses to whole-grains are different for each recipe.

 The insulin results did not support our hypothesis. When matched for available CHO, insulin incremental AUCs for 11-grain and sprouted-grain breads were greater than sourdough and white breads (Table 3). This is consistent with the glucose data for sprouted-grain and sourdough breads, but does not explain the glucose result for 11-grain bread, suggesting that glycemia does not always predict insulinemia. In the present study, acute ingestion of 50 g available CHO from whole-grain and sprouted-grain breads did not improve insulinemia or insulin sensitivity (as assessed by calculation of ISI) compared to white bread. Limited literature is available on acute intervention and the results from epidemiologic [4, 5, 7] and chronic interventional [9, 10] studies suggest that any positive effect of whole-grain food intake on insulinemia and insulin sensitivity is only apparent after a chronic intervention. These findings may help explain the lack of positive effect of acute ingestion of whole-grain breads on insulinemia and insulin sensitivity in our study.

 It should be noted that the magnitude of the glucose and insulin (Tables 3 and 4) responses to the sourdough bread was similar in parts 1 and 2 of the study, respectively, and that these data are consistent with those reported previously from our laboratory [18]. While we previously showed that sourdough bread resulted in a more favourable postprandial response compared with whole-wheat bread [18], the breads were all prepared in the laboratory. In the present study, the comparison was with whole-grain (not whole-wheat) breads that were commercially prepared. In the former investigation [18], the breads were all administered to control for available CHO and thus subjects ingested different amounts of breads. In part 2 of the current study, matching the treatments for volume of bread consumed resulted in a large difference in available CHO content among the breads. The lower glucose and insulin incremental AUCs for the whole-grain breads compared to those of sourdough bread can be attributed to the lower available CHO and greater dietary fiber content of the whole-grain treatments.

 Incretins are potent insulin-releasing hormones that play an important role in glucose homeostasis. Previously we observed that sourdough bread resulted in lower GLP-1 response [18]. In part 1 of the present study, GIP responses to the ingestion of 50 g available CHO of the breads did not differ significantly among the test breads. However, in part 2, ingestion of equal amounts of the test breads resulted in significantly lower GIP incremental AUC for 11-grain and 12-grain breads compared to white breads, a result that may be attributed to the lower available CHO content of these breads. However, the GIP response to sprouted-grain bread, with the lowest available CHO content, was not lower than those to white bread. In both parts 1 and 2, the insulin responses did not appear to follow that of the incretins. These findings suggest that postprandial responses for different whole-grain breads are complex and cannot be explained only by the available CHO content.

Consistent with our previous study [18], in part 1 of the present study, overall GLP-1 response to sourdough bread was significantly lower than 11-grain, sprouted-grain, and white breads. Consistently, insulin response to sourdough bread in part 1 was significantly lower than 11-grain and sprouted-grain breads. Bakhoj et al. [33] reported lowered postprandial GIP responses to the ingestion of Einkorn honey-salt leavened and whole-grain breads compared to the conventional yeast bread and proposed that this was due to an increased level of organic acids (based on reduction of the pH in the dough). Dietary fiber has also been shown to increase GLP-1 secretion in rats [34] and dogs [35]. A study by Massimino et al. [35] found that highly fermentable dietary fibers were more potent stimulators of GLP-1 secretion compared to low fermentable fibers. Given that the fermentable, insoluble fibre content was greatest in the sprouted-grain and lowest in the sourdough bread, it is reasonable to speculate that the GLP-1 response observed in the present study may in part be influenced by insoluble fiber content of the breads. Lastly, it is important to note that a more refined study of incretin dynamics in an animal model may better characterize the transient postprandial nature of these peptides taking into account their relatively short half-lives. In our study, however, we were ethically constrained and only able to draw a certain number of samples that were mixed venous in nature thus why we opted to examine the incretin response as incremental AUC.

Overall, the results of the current investigation suggest that glucose metabolism is complex and multifactorial. The simple model of glucose stimulated insulin secretion, and incretins regulating postprandial insulin release does not always apply. Additionally, GIP and GLP-1 do not respond in a similar manner with respect to the CHO ingested. In our previous [18] and present studies, we showed that the nature of the bread consumed has an impact on glucose, insulin, and incretin responses, but the mechanism is complex and requires further investigation.

To our knowledge, this is the first study to compare postprandial responses to ingestion of various breads delivering an identical amount of available CHO (thus different masses) with the postprandial effect of ingestion of a fixed portion size (thus same volume, but different amounts of available CHO) of the same breads in overweight/obese men. It appears that bread volume and fiber content may play a role but are not the dominant factors in determining the metabolic responses to the breads, as in part 2 of the study, 11-grain, sprouted-grain, and 12-grain breads, with similar volume and fiber content, induced different results in almost every measure. These results suggest that the nature of the ingredients is an important factor influencing the metabolic responses to the breads. Lack of difference between 11-grain and white breads was unexpected but it may be that any positive impact of 11-grain on glucose metabolism only occurs after a chronic dietary intervention.

## 5. Conclusion

 While the study is limited due to its applied nature (i.e., employing commercial breads and a somewhat heterogeneous subject set), this is also a strength in terms of applying the findings to the lifestyle of society. Despite the variation that these factors produced, the investigation demonstrated that sprouted grain bread attenuated the glycemic response when both portion size and available carbohydrate were controlled for and that, generally, the whole-grain breads did not have what could be interpreted as beneficial, metabolic responses.

## Figures and Tables

**Table 1:** Nutrient composition of the test breads delivering 50 g available CHO (part 1)^1^.

	11-grain	Sprouted-grain	Sourdough	12-grain	White
Total bread (g)	151.0	157.2	107.3	122.2	110.3
Available CHO (g)^2^	50.0	50.0	50.0	50.0	50.0
Energy (kcal)	320.2	336.4	277.9	317.8	273.7
Starch (g)	44.9	46.3	45.4	42.5	43.6
Total sugars (g)	5.1	3.6	4.5	7.4	6.4
Soluble fiber (g)	0.9	0.6	0.3	1.1	0.3
Insoluble fiber (g)	11.9	11.4	4.9	9.9	4.6
Dietary fiber (g)	12.8	12.1	5.2	11.0	4.9
Protein (g)	16.9	22.3	9.0	12.6	9.8
Fat (g)	3.1	2.9	4.3	5.2	3.6

^1^Test breads were analyzed by Laboratories of Canada Incorporated (ILC) in Tillsonburg, ON.

^2^Available CHO was calculated using this formula: starch + total sugar.

**Table 2:** Nutrient composition of the test breads delivering a consistent portion size (Part 2)^1^.

	11-grain	Sprouted-grain	Sourdough	12-grain	White
Total bread (g)	107.3	107.3	107.3	107.3	107.3
Available CHO (g)^2^	35.5	34.0	50.0	43.8	48.6
Energy (kcal)	227.4	229.6	277.9	278.9	266.0
Starch (g)	31.2	31.6	45.4	37.3	42.3
Total sugars (g)	4.3	2.4	4.5	6.5	6.2
Soluble fiber (g)	0.6	0.4	0.3	0.9	0.3
Insoluble fiber (g)	8.4	7.8	4.9	8.6	4.5
Dietary fiber (g)	9.1	8.2	5.2	9.6	4.8
Protein (g)	12.0	15.2	9.0	11.0	9.5
Fat (g)	2.2	2.0	4.2	4.6	3.5

^1^Test breads were analyzed by Laboratories of Canada Incorporated (ILC) in Tillsonburg, ON.

^2^Available CHO was calculated using this formula: starch + total sugar.

**Table 3:** Incremental area under the curve for blood glucose, serum insulin, plasma GIP and GLP-1 after ingestion of 50 g available CHO of the test breads for 180 min (part 1)^1, 2^.

	11-grain	Sprouted-grain	Sourdough	12-grain	White
Glucose (mM/L·min)	0.64^a^ ± 0.04	0.22^b^ ± 0.17	0.66^a^ ± 0.16	0.26^bc^ ± 11.0	0.51^ac^ ± 0.17
Insulin (nM/L ∗ 180 min)	31.6^a^ ± 6	30.4^a^ ± 5	21.4^b^ ± 3.3	25.9^ab^ ± 6.8	24.1^b^ ± 4.5
GIP (nM/L ∗ 180 min)	3.2 ± 0.4	3.7 ± 0.3	3.6 ± 0.4	3.6 ± 0.4	3.3 ± 0.2
GLP-1^3^ (nM/L ∗ 180 min)	0.58 ± 0.23	0.57 ± 0.32	−0.04 ± 0.16	0.38 ± 0.2	0.41 ± 0.31

^1^All values are mean (±SEM); (*n* = 12) except for GLP-1 (*n* = 11) because of technical problems.

^2^Mean values within a row with different superscript letters were significantly different (*P* < 0.05).

^3^Data was log-transformed prior to statistical analysis and is presented as the geometric mean ± SEM.

**Table 4:** Incremental area under the curve for blood glucose, serum insulin, plasma GIP and GLP-1 responses to the ingestion of set amount of the test breads for 180 min (part 2)^1, 2^.

	11-grain	Sprouted-grain	Sourdough	12-grain	White
Glucose (mM/L·min)	0.31^a^ ± 0.12	0.17^a^ ± 0.15	0.72^b^ ± 0.19	0.41^a^ ± 0.11	0.46^a^ ± 0.14
Insulin (nM/L ∗ 180 min)	16.2^ac^ ± 2.1	12.7^ac^ ± 1.9	21.5^b^ ± 2.7	16.8^a^ ± 2.4	18.1^bc^ ± 3.4
GIP (nM/L ∗ 180 min)	2.7^a^ ± 0.3	3.1^ab^ ± 0.3	3.5^bc^ ± 0.3	3.3^ac^ ± 0.4	4.0^b^ ± 0.7
GLP-1^3^(pM/L ∗ 180 min)	0.48^ab^ ± 0.2	0.83^a^ ± 0.3	−0.05^b^ ± 0.1	−0.19^b^ ± 0.5	0.07^ab^ ± 0.3

^1^All values are mean (±SEM); (*n* = 11) except for GLP-1 (*n* = 10) because of technical problems.

^2^Mean values within a row with different superscript letters were significantly different (*P* < 0.05).

^3^Data was log transformed prior to statistical analysis and is presented as the geometric mean ± SEM.
